# Mapping knowledge structure and themes trends of biodegradable Mg-based alloy for orthopedic application: A comprehensive bibliometric analysis

**DOI:** 10.3389/fbioe.2022.940700

**Published:** 2022-08-09

**Authors:** Zitian Zheng, Wennan Xu, Yanan Xu, Qingyun Xue

**Affiliations:** ^1^ Department of Orthopedics, Beijing Hospital, National Center of Gerontology, Institute of Geriatric Medicine, Chinese Academy of Medical Sciences, Beijing, China; ^2^ Fifth School of Clinical Medicine, Peking University, Beijing, China

**Keywords:** Mg-based alloy, biodegradable metal material, orthopedics, bibliometric analysis, visualized study

## Abstract

**Background:** Since Lambotte and Payr first studied Mg-based alloys for orthopedics in 1900, the research of this field has finally ushered in vigorous development in the 21st century. From the perspective of quantitative analysis, this paper clearly demonstrated the global research trend from 2005 to 2021 by using bibliometrics and scientometric analysis.

**Methods:** We obtained the publications from the Web of Science Core Collection (WoSCC) database. The bibliometric and scientometric analysis was conducted by using R software, CiteSpace software, VOSviewer software, Pajek software and Microsoft Excel program.

**Results:** In total, 1921 publications were retrieved. It can be found that the number of publications is gradually increasing year by year. We can find that the most prolific countrie, institution and researcher are China, Chinese Academy of Sciences and Zheng Yufeng, respectively. The most influential journals in this field are Acta Biomaterialia and Biomaterials, with 16,511 and 12,314 total citations, respectively. By conducting the co-cited documents-based clustering analysis, 16 research hotspots and their representative studies have been identified. Besides, by conducting analysis of keywords, we divided the keyword citation bursts representing the development of the field into three stages.

**Conclusion:** The number of researches on the biodegradable Mg-based alloys increased sharply all over the world in the 21st century. China has made significant progress in biodegradable Mg-based alloy research. More focus will be placed on osteogenic differentiation, fabrication, graphene oxide, antibacterial property, bioactive glass and nanocomposite, which may be the next popular topics in the field.

## 1 Introduction

Since the 1960s, people have studied and screened traditional industrial materials based on biocompatibility (Navarro et al., 2008), and developed the first generation of biomedical materials characterized by biological inertia and *in vivo* stability. In the mid-1980s, a variety of bioactive materials including bioactive glass (El-Ghannam et al., 1999), bioceramics (Hench, 1991) and absorbable suture (Bezwada et al., 1995) began to be widely used.

The first- and second-generation medical implants, such as such as 316L stainless steel, pure titanium and Ti-6Al-4V Alloy and cobalt chromium molybdenum alloy, are still widely used in orthopedics. However, Since the 21st century, the third generation of medical materials that can induce cell proliferation, differentiation, and the synthesis and assembly of extracellular matrix are in the ascendant (Navarro et al., 2008; Holzapfel et al., 2013). The third generation of medical materials, namely biodegradable materials, is the combination of two independent concepts of bioactive materials and degradable materials (Yang et al., 2018).

As the third-generation biomedical material with bright clinical application prospects, magnesium alloy has the advantages of avoiding secondary surgery and promoting tissue regeneration (Chen et al., 2018). The research on the mechanical properties, degradation properties and biocompatibility (Wang et al., 2022) of magnesium alloys is a research hotspot that researchers all over the world are committed to, but there is still a long way to go before the clinical application of magnesium alloy products (Azadani et al., 2022). The surge of researches and literatures makes this field complicated and profound. Bibliometrics can review and prospect this field from a clear, objective and comprehensive perspective (Kumar and Goel, 1007).

Because of the uncontrollable degradation rate and hydrogen production, the research on magnesium alloys in the 20th century fell into a stagnant period for a long time, and did not enter a rapid development stage until the 21st century. Researchers are mainly making efforts to optimize the design of alloy composition, improve the manufacturing technique, regulate the modification of microstructure and surface (Li et al., 2014). Besides, researchers explored the mechanism promoting osteoconduction, osseointegration and osteogenic activity (Yoshizawa et al., 2014) through various methods both *in vitro* and *in vivo* (Witte et al., 2008). With the progress of industrial manufacturing technique and computer science, new technology, such as additive laser manufacturing (ALM 3D Printing) (Shuai et al., 2019), finite element analysis, boundary element method ^15^and atomic layer deposition, makes this field full of vitality.

The development of medical magnesium alloys is a long process**:**

a) From the perspective of composition, the development process is roughly as follows: (1) alloying (2) microalloying (Stanford et al., 2008) (3) plain materials (Yang et al., 2017) (4) high purification;
b) From the perspective of structure, the development is roughly as follows: (1) multiphase and multicrystalline (Bottger et al., 2006) (2) single phase (3) amorphous (Wu et al., 2017).
c) The development of nano materials has accelerated the research and development of magnesium alloys. In addition, the composition of magnesium alloys is also changing, from Mg-Al, Mg-Zn and Mg-Ca alloys to Mg-Re alloys with rare earth elements (Luo et al., 2020), as well as Mg-Sr and Mg-Sc alloys which have great application potential (Wang et al., 2014).

### 1.1 The implication of the state-of-art review

According to our systematic review on the Clarivate Analytics Web of Science (WOS) database (Harzing and Google Scholar, 2016), the reviews on magnesium alloys for orthopedic use in recent 5 years have been included in Supplementary Table S1. These reviews have summarized and revealed the great potential and bright prospects of magnesium alloys for orthopedic applications from various angles, but there was no bibliometric analysis regulating Mg-based alloy for orthopedic applications (Latest search update: 10 Feb 2022).

The previous reviews included the valuable opinions put forward by experts from their own point of view. If we can make the systematic analysis in the form of bibliometrics, closely and rigorously review the relevant variables of literature in this field, and build a literature network by means of metrology and statistics (Thelwall, 2008), we can further accelerate the process of research, development and application of magnesium alloys for orthopedics (Ninkov et al., 1007).

Bibliometric analysis is based on the global literature, using mathematics, statistics and other methods, to study the distribution pattern, quantitative relationship and evolution law of the literature, then to explore and demonstrate the structure, characteristics, distribution and development trend of research in the field (Zhao et al., 2022). Bibliometrics research includes analysis of distribution of literatures by year, co-citation analysis (Yang et al., 2019), co-authorship network analysis, and text mining (Moral-Munoz et al., 2020).

### 1.2 Research questions and intended contribution of the study

Keeping in mind the research gap derived from the above analysis, the study focuses on one of the essential research questions:

“Do we need any Bibliometric Analysis of the Biodegradable Mg-based Alloy for Orthopedic Application?”

The answer to this research question is yes; as a rapidly developing and promising field, the global trend of magnesium alloy research is still unclear. So, it was of necessity to adopt bibliometric methods to clearly show the development process, current research status and future trend of this field from a qualitative and quantitative point of view (Cobo et al., 2011; Kumar et al., 2022). In this case, our research is committed to helping young researchers gain an exhaustive picture of the current research status in this field and enables them to determine their own research directions, seek the support of research platforms and institutions that may be helpful to them, and quickly retrieve classic literature. Our research will increase the overall system output of biodegradable Mg-based alloys for orthopedics to a certain extent.

### 1.3 Objectives of the present review

Based on the above research gap and the expected contribution, our research is based on the following research objectives:

1) To conduct an extensive bibliometric analysis of biodegradable Mg-based alloy for orthopedic application.
2) To put forward the research prospect of biodegradable Mg based alloy for orthopedic application, and describe its possible application scope in the future.

With the help of the research tools and software discussed in the “Materials and Methods” section, this study organized a comprehensive bibliometric and statistical analysis of magnesium alloys according to the trend of literature publication, the co-authorship between countries, institutions, researchers, and the co-citation of journals, references and keywords.

## 2 Materials and methods

**FIGURE 1:**
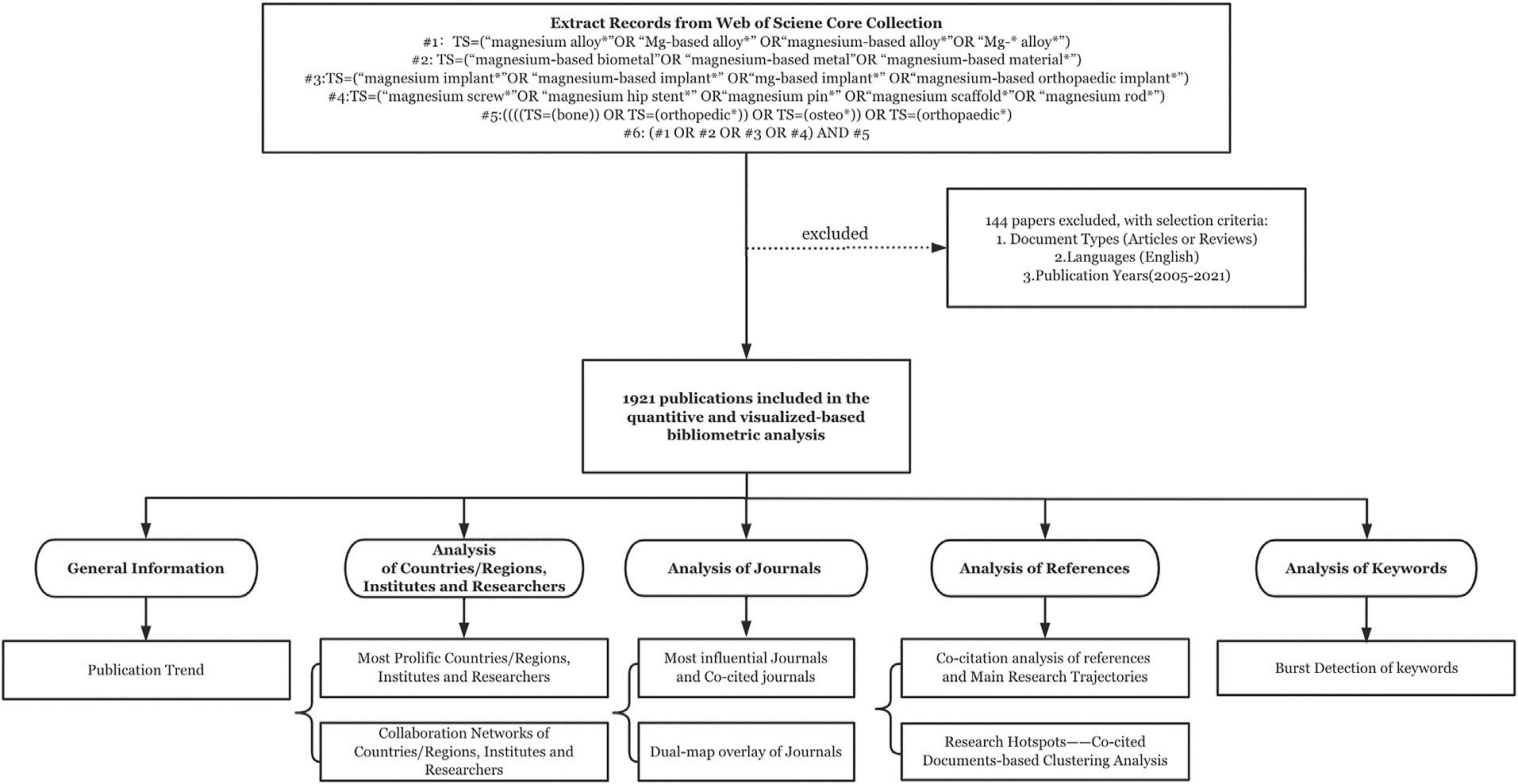
The work flow diagram.

The comprehensive search of data was conducted on the Science Citation Index Expanded (SCI-Expanded) of the Clarivate Analytics Web of Science Core Collection (WoSCC) on a single day to avoid the discrepancies due to daily database updates (Mongeon and Paul-Hus, 2016). WoSCC is an elaborate collection of high-quality academic peer-reviewed literature published worldwide (Mongeon and Paul-Hus, 2016), which provides various retrieval methods and download paths for bibliometric analysis. The search phrase was shown in Figure 1. Because there was almost no literature published in this field before 2005, we set the search timespan as 2005-2021, which is a long enough time period to reflect the development trends. The present analysis was concerned with only two types of documents, articles and reviews published in the English language, and no species restrictions were imposed. Finally, 1921 records were finally retrieved. Then “Full Records and Cited References” were selected in the exported content of the WOS records, “Pure Text” was selected in the file format. The detailed screening and work flow chart are shown in Figure 1.

## 3 Data analysis

We mainly employed four analytic softwares and Microsoft Excel program to perform bibliometric analysis.

Briefly, we used the “bibliometrix” package (version 3.0.3, http://www.bibliometrix.org) installed in R software 4.0.3, which provides a web-interface for bibliometric analysis. (Aria and Cuccurullo, 2017).

CiteSpace software (version 5.7 R5W, https://citespace.podia.com/courses/download) and VOSviewer software were used for the quantitative analysis and mapping knowledge domain so that readers can directly visualize the evolution and development process (van Eck and Waltman, 2010). More information about the CiteSpace parameters and the mechanism of generation of knowledge network is available in Professor Chen’s articles. (Chen, 2004; Chen, 2020).

Generally speaking, the figures generated by the bibliometric software consist of nodes and lines. Through the labels attached to the nodes, we can identify the various elements represented by the nodes, including articles, authors, institutions, keywords, etc. Lines represent the connections within the elements, including co-author analysis, co-citation analysis, co-occurrence analysis, etc. And the size of the nodes represents the number of publications, citations, or occurrences.

In addition, analysis of main research trajectories was conducted by Pajek software (Mrvar and Batagelj, 2016), a software program for the analysis and visualization of the main paths in large networks. After carefully investigating these documents in the main paths (Liu and Lu, 2012), we can efficiently comprehend the major research trajectories in the field, which facilitates us to better grasp the current hotspots and predict the future trends.

## 4 Results

### 4.1 Distribution of articles by publication years

**FIGURE 2:**
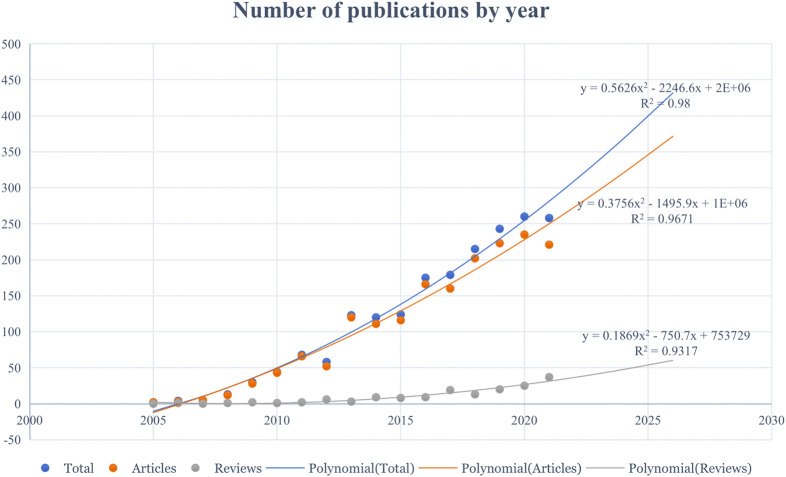
Trends in publications from WOS (2005–2021) by year in the field of the biodegradable Mg-based alloy for orthopedic application and the corresponding polynomial fitted curves.

Accessibility to Web of science, one of the largest citation databases in the world, was obtained through Peking University library. We retrieved a total of 1921 documents related to Mg-based alloy for orthopedic application. As is shown in Figure 2, We can see that the number of articles is gradually increasing by year. Polynomial model fitting revealed significant correlations between the publication year and the publication output (the coefficients of determination (R2) were 0.98, 0.967, and 0.932 for total documents, articles, and reviews, respectively). On the basis of polynomial curve fitting, the publication output is expected to reach approximately 400 in 2025, comprising 350 articles and 50 reviews.

### 4.2 Active countries/regions, institutes and researchers

**FIGURE 3:**
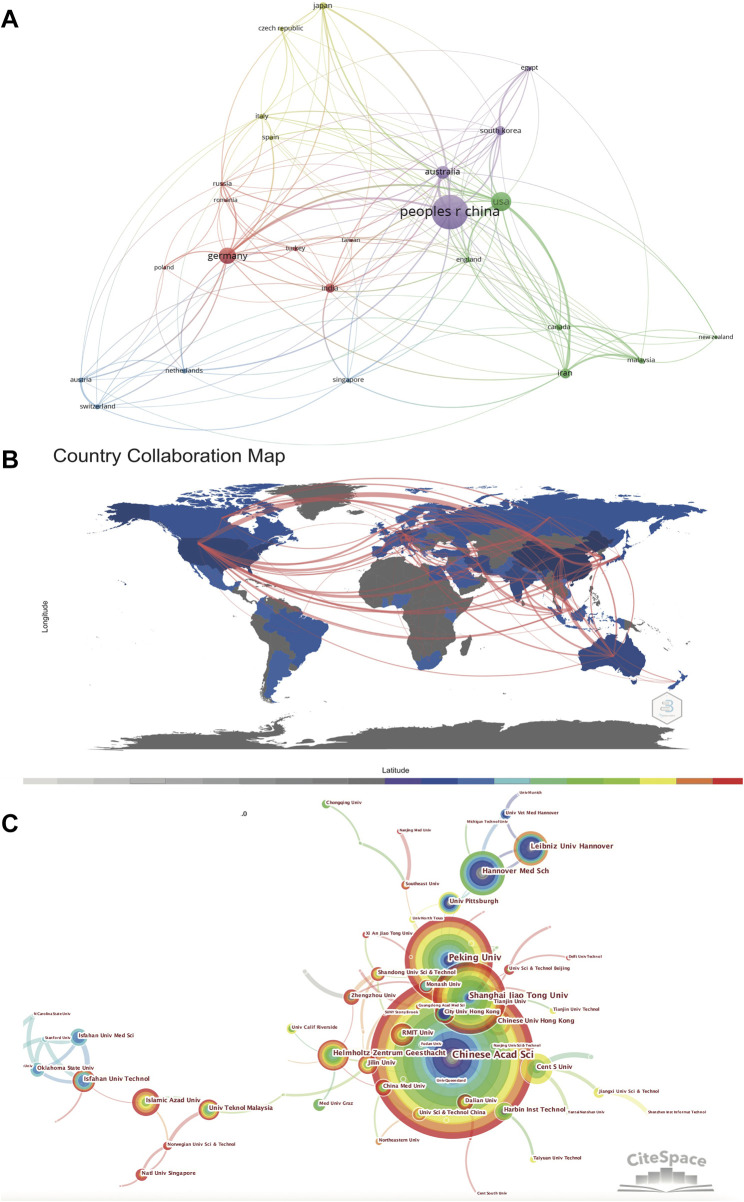
**(A)** Country Collaboration Network generated by the VOSviewer software **(B)** the Country Collaboration plotted on the world map. **(C)**Collaboration network of institutions generated by the Citespace software.

Figure 3(A,B) and Table 1 show the cooperation networks among countries and the details of the 10 most productive countries, respectively. The articles published by China had the most citations (30,933), followed by Germany (17,266), the United States of America (United States) (9,109) and Australia (5,269). It can be seen that the leading countries in this field are strong manufacturing countries with solid industrial systems. Since the research of magnesium alloys for orthopedics is multidisciplinary research of medicine and material science, the development of technology and engineering is of vital importance to the progress of medicine and biology.

**TABLE 1:** The top 10 prolific countries from 2001 to 2021.

Rank	Country	Documents	Citations	Average article citations	Total link strength
1	Peoples R China	855	30,933	36.18	195
2	United States	268	9,109	33.99	204
3	Germany	256	17,266	67.45	139
4	India	120	2,574	21.45	37
5	Iran	107	2,338	21.85	81
6	Australia	89	5,269	59.20	70
7	SouthKorea	89	2,851	32.03	63
8	Japan	51	1946	38.16	48
9	Malaysia	51	1,225	24.02	60
10	Canada	40	2,168	54.20	50

The top 20 institutes are listed in Table 2. Collaborations among these institutes were shown in Figure 3C. Chinese academy of sciences had the most publications (161), followed by Peking University (99), Shanghai Jiao Tong University (84) and Hannover Medical School (68). Most of these institutions are world-renowned research institutes, with prominent positions in the history of biomaterial research and development. It can be found that an effective international cooperation network has been formed in this field. Chinese research institutions such as Chinese Academy of Sciences, Peking University and Shanghai Jiao Tong University have established close cooperative relationship with German research institutions such as Hannover University, Helmholtz-Zentrum Geesthacht and American research institutions such as University of Pittsburgh (Li et al., 2016), the University of Tennessee and the Ohio State University (Jia et al., 2021). Focusing on the academic achievements of these prolific research institutions can help us better understand the development of biodegradable Mg-based alloys.

**TABLE 2:** The top 20 prolific institutions from 2001 to 2021.

Rank	Affiliations (affiliation name disambiguation)	Number of publications	Citations	Total link strength
1	Chinese Acad SCI	161	8,696	230
2	Peking Univ	99	7,747	175
3	Shanghai Jiao Tong Univ	84	3,857	83
4	Hannover Med SCH	68	10,674	114
5	Leibniz Univ Hannover	60	4,353	102
6	Helmholtz Zentrum Geesthacht	46	1,374	29
7	Cent S Univ	44	1,645	58
8	Univ Teknol Malaysia	38	838	37
9	Zhengzhou UNIV	38	1,578	37
10	City Univ Hong Kong	37	2,431	47
11	Harbin inst Technol	36	1,557	45
12	Isfahan Univ Technol	36	1,172	58
13	Islamic Azad Univ	34	581	41
14	Univ Pittsburgh	34	1945	19
15	Univ Vet Med Hannover	31	1,530	64
16	Jilin Univ	30	469	22
17	Chinese Univ Hong Kong	29	2,208	61
18	Tianjin Univ	29	589	45
19	China Med Univ	28	1,573	38
20	Med Univ Graz	28	1,391	36

**FIGURE 4:**
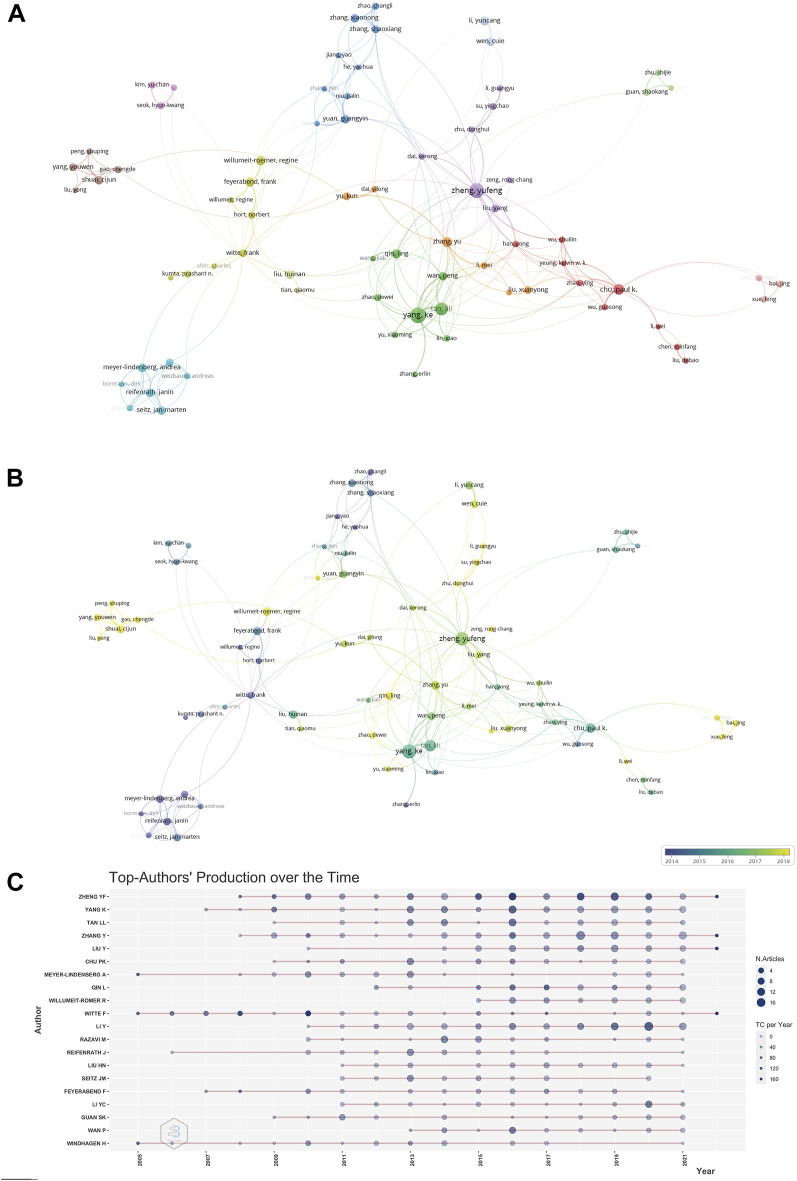
**(A)** Collaboration network of researchers generated by the VOSviewer software. **(B)** Collaboration overlay visualization of researchers generated by the VOSviewer software. **(C)** The top twenty prolific researchers in the field and their publications over time. The larger the node, the more articles published. The deeper the color, the more citations.

A total of 520 publications were from the top 15 authors, accounting for 27.07% of all publications in this field. The most influential author is Zheng Yufeng, with 85 papers and 7,421 citations, followed by Witte Frank from Charite universitatsmedizin Berlin, who laid the foundation for the development of this field in the early stage, as well as Yang Ke from the Chinese Academy of Sciences and Feyerabend Frank from Helmholtz Association. Table 3 and Figure 4C show the details of the top active researchers in this field and their productions over time, respectively. Collaborations among these authors were shown in Figures 4A, B. It can be found that the three most prolific researchers, namely Zheng Yufeng from Peking University and Tan Lili, Yang Ke from the Institute of Metals Research, CAS have close collaboration with each other, and they have also continued to produce high-quality research papers in recent years. From Figures 4B,C, it can be found that Prof. Witte Frank was extremely prolific at the initial stage of the Mg-based alloy research, laying a solid foundation for the prosperity of the discipline. In addition, we found that the international cooperation between top scholars has been established. For example, Professor Qin Ling and Professor Wang Jiali from the Chinese University of Hong Kong, Professor Zheng Yufeng from Peking University, Professor Witte Frank from Charite Universitatsmedizin Berlin and Zhao Dewei from Dalian University reviewed and summarized the current situation of clinical transformation of magnesium alloys in 2018, and obtained huge influence (Zhao et al., 2017a). The cooperative relationship between more researchers can be seen from Figure 4. In Figure 4A, the researchers are divided into clusters of different colors, and the researchers in each cluster have a relatively close cooperative relationship. For example, the green cluster mainly reflected the cooperative relationship between scholars from the Institute of Metals Research, CAS and Chinese University of Hong Kong (Yan et al., 2018). In addition, the change of colors in the overlay visualization in Figure 4B reflects the active time period of researchers (Carley et al., 2017). The lighter the color, the more recent the researchers have been active in this field. Equally non negligible is the significant role of female researchers, with excellent contributions from Prof. Tan Lili from the Institute of metals Research, CAS. (Tan et al., 2013).

**TABLE 3:** The top 10 prolific authors ranked by the citations.

Rank	Author	Documents	Citations	Total link strength	H-index	Affiliation
1	Zheng, Yufeng	85	7,421	238	84	Peking University
2	Yang, Ke	63	3,696	190	46	Chinese Academy of Sciences
3	Tan, Lili	45	1,694	134	39	Chinese Academy of Sciences
4	Chu, Paul K	32	1866	86	109	City University of Hong Kong
5	Shuai, Cijun	32	1,283	126	40	Central South University
6	Witte, Frank	31	6,789	21	34	Charite Universitatsmedizin Berlin
7	Willumeit, Regine	30	673	41	49	Helmholtz Association
8	Feyerabend, Frank	27	3,324	63	33	Helmholtz Zentrum Geesthacht
9	Zhang, Yu	27	618	121	32	Guangdong Academy of Medical Sciences
10	Qin, Ling	25	1,523	73	61	Chinese University of Hong Kong
11	Razavi, Mehdi	25	1,042	84	23	University of Central Florida
12	Reifenrath, Janin	25	1,032	98	23	Hannover Medical School
13	Seitz, Jan-Marten	25	848	70	24	University of Hannover
14	Yuan, Guangyin	25	795	89	40	Shanghai Jiao Tong University
15	Meyer-lindenberg, Andrea	23	1,086	91	97	League of European Research Universities/Hannover Medical School/University of Munich

### 4.3 Journals

**FIGURE 5:**
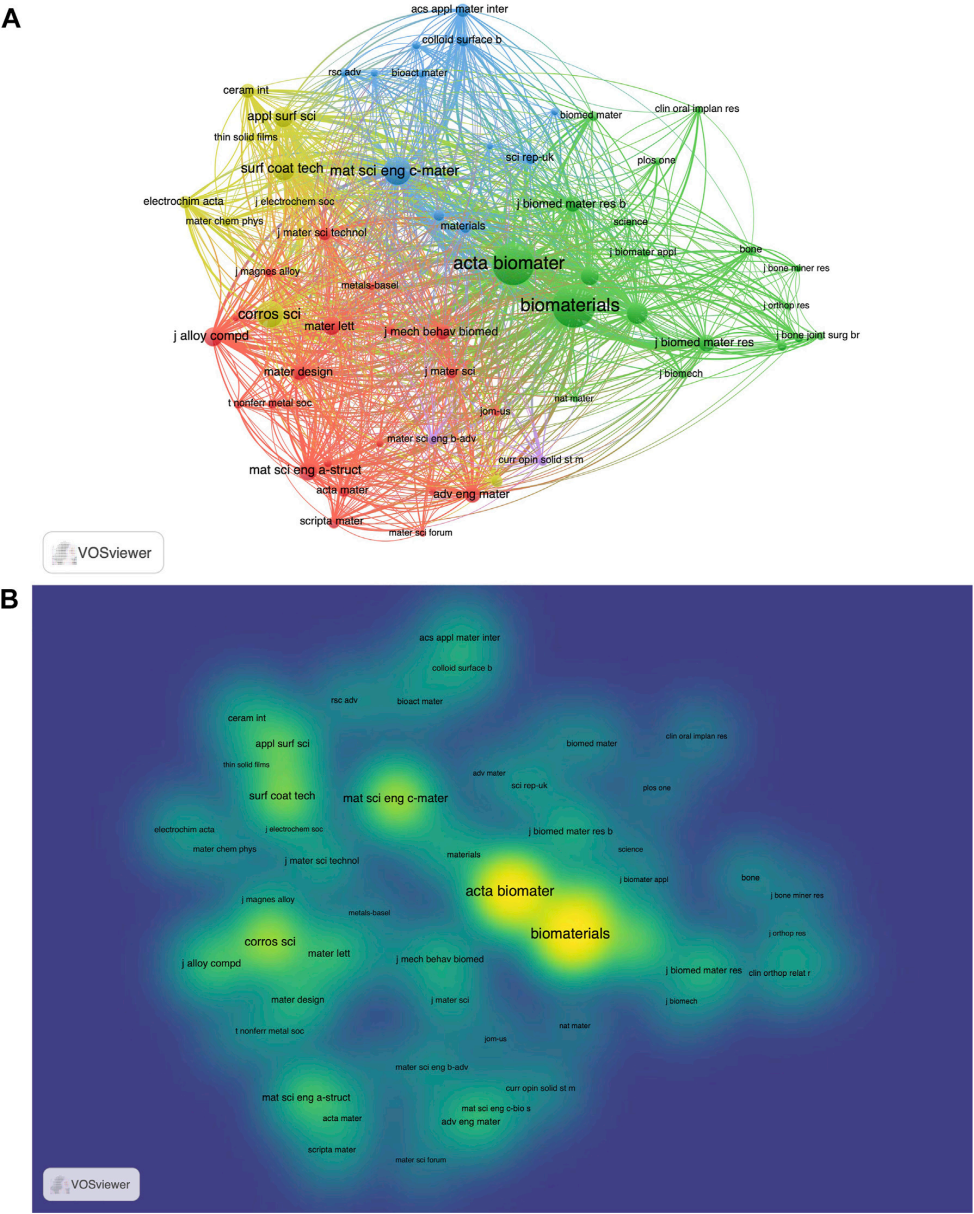
**(A)** Cluster visualization of the journal co-citation analysis generated by the VOSviewer software. Each node represents a journal, and the size of each circle is determined by the co-citations of the journal. **(B)** Density visualization of the journal co-citation analysis generated by the VOSviewer software.

The reference relationship of academic journals represents the knowledge exchange in the research field, in which the cited papers form the Frontier of knowledge, and the cited papers form the basis of knowledge. The top 15 journals were presented in Table 4. Visualization of the journal co-citation analysis was shown in Figure 5 (Hu et al., 2011). The journal with the largest total citations is Acta Biomaterialia (total citations = 16,511) while the journal with the largest number of publications is Materials Science & Engineering C-Materials for Biological Applications (Number of Publications = 147). The journals in the list of Table 4 may be the main channels of publications for future discoveries in this field.

**TABLE 4:** The top 15 journals with the most publications ranked by the H-index.

Rank	Source titles	Documents	Citations	Total link strength	Impact factor	WoS quartiles	H Index
1	Acta biomaterialia	145	16,511	5,101	8.947	Q1	207
2	Biomaterials	26	12,314	3,382	12.479	Q1	397
3	Materials Science Engineering C: Materials for Biological Applications	147	5,069	2,507	7.328	Q1	145
4	journal of biomedical materials research part A	38	2,622	1,320	4.396	Q2	159
5	Applied Surface Science	49	2,141	749	6.707	Q1	204
6	Journal of Materials Science and Technology	35	1,646	640	8.067	Q1	81
7	Surface and Coatings Technology	78	1,596	907	4.158	Q2	182
8	Journal of Alloys and Compounds	52	1,511	794	5.316	Q2/Q1	185
9	Journal of the Mechanical Behavior of Biomedical Materials	53	1,497	703	3.902	Q2/Q3	99
10	Materials	53	1,351	693	3.623	Q2/Q1	128
11	Journal of Biomedical Materials Research Part b-Applied Biomaterials	37	1,291	821	3.368	Q2/Q3	116
12	Materials and Design	29	1,180	552	7.991	Q1	187
13	Journal of Materials Science-Materials in Medicine	37	1,086	691	3.896	Q2/Q3	133
14	Scientific Reports	21	971	367	4.38	Q1	242
15	Ceramics International	32	817	383	4.527	Q1	126

**FIGURE 6:**
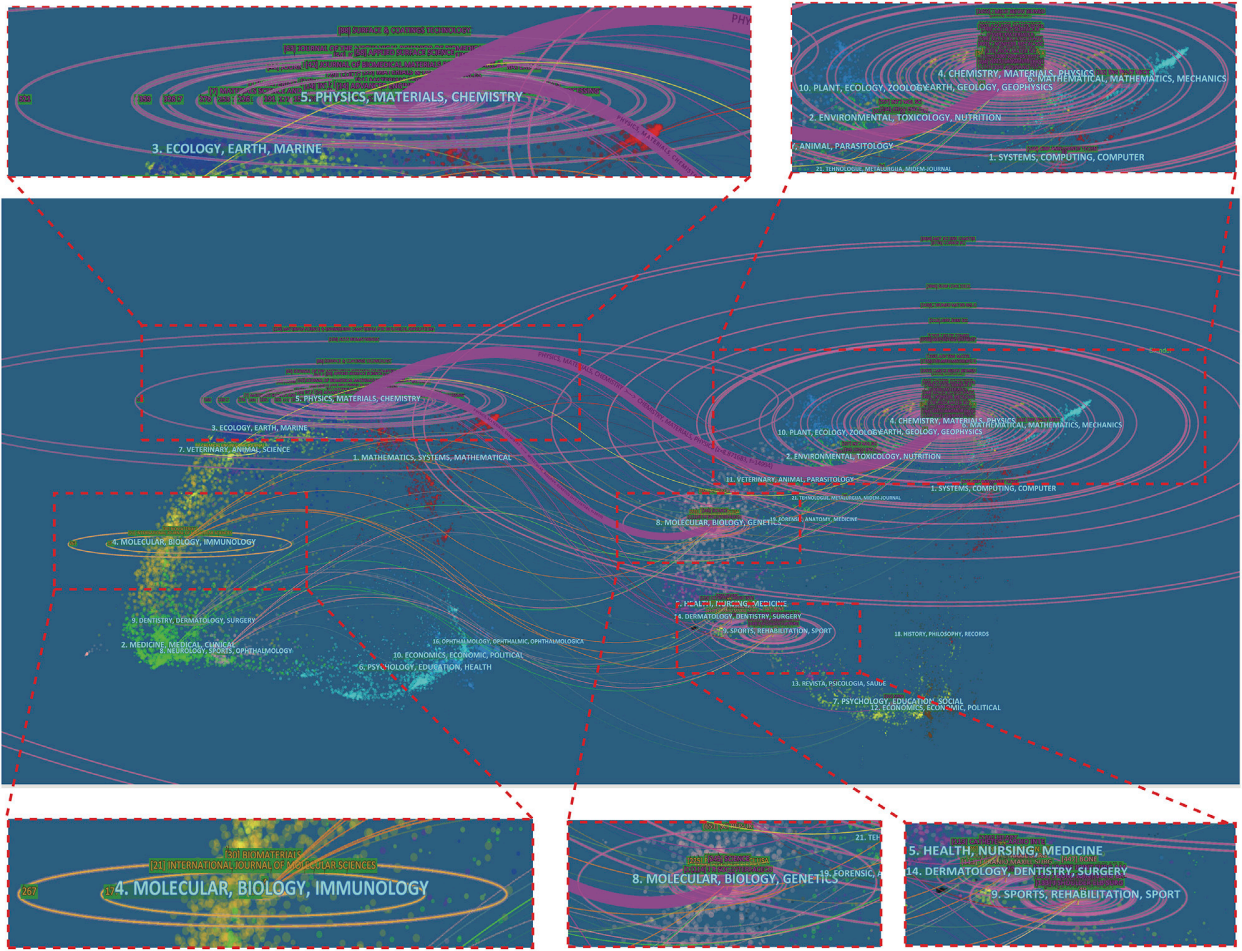
The dual-map overlay of journals on Mg-based alloy for orthopedic application.

The dual-map overlay of journals, a new method of publication portfolio analysis invented by professor Chen Chaomei, was shown in Figure 6, with the citing journals on the left side, cited journals on the right side, and the colored paths indicate the citation relationships. The width of the connecting paths is proportional to the frequency of z-score-scale citation. The journals are grouped into clusters by adopting the Blondel algorithm to identify the major research disciplines. It can be found that studies, published in Physics/Materials/Chemistry journals, usually cite the studies which published in Chemistry/Materials/Physics and Molecular/Biology/Genetics journals. This indicates that in the research of biomaterial, the development of chemistry effectively supports the progress of physics and materials science. And the development of materials science, physics and chemistry has been oriented towards molecular, physiology and genetics. And it is easy to find that the third most enriched cluster of journals is Molecular/Biology/Genetics, which is widely cited in the other clusters including sports, rehabilitation, physics and materials. More information about the representative citing and citied journals in each cluster can be detected in Supplementary Table S2.

### 4.4 Reference

Analysis of references is one of the most important indicators of bibliometrics. We mainly conduct the analysis of references from two aspects.

1) Co-citation analysis of references and Main research trajectories in the biological Mg-based alloy research field.

**FIGURE 7:**
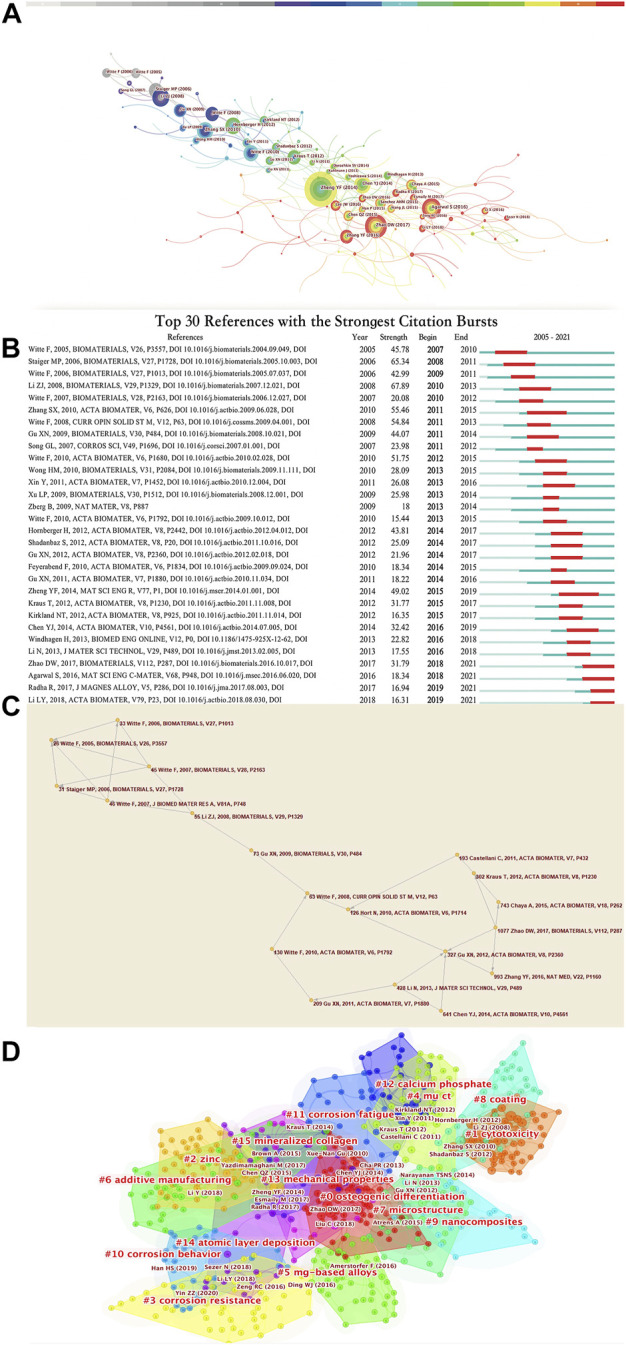
**(A)** Intellectual base of research on Mg-based alloy for orthopedic application. This figure is arranged in chronological order from left to right. It can be seen that the influential literature has gradually increased in recent years, and more links have been generated. **(B)** The top 30 references with the strongest citation bursts. The red bars indicate the duration of the burst, namely, the time period when the keyword is highly frequently cited, while the green bars represent the time period when the keyword is less infrequently cited. **(C)** The research main path during 2005–2021. **(D)** Cluster visualization of the co-citation network of references *via* Citespace, together with the representative references of the generated clusters.

Frequently cited literatures usually have great influence in the relevant research fields. As is shown in Figure 7A, A network of co-cited references was constructed to demonstrate the most significant studies. The parameters were set as follows: # Years Per Slice = 1, Top N% = 1, pruning algorithm was adopted. A network map with 175 nodes, and a network density of 0.0187 was obtained. The most cited articles are shown in Supplementary Table S3. These articles are obviously the most influential articles in this field. However, because of the snowball effect of the accumulation of citations of the references, the citation burst algorithm of Citespace was employed to show the most influential documents cited frequently in different time period, and show the top 30 references with the strongest citation bursts as Figure 7B. These articles have had a significant impact in their respective time periods. The time period represented by the outer color circle of the nodes corresponding to each reference in Figure 7A represents the burst time duration in Figure 7B. It can be seen that the references with the strongest citation bursts are Li ZJ, 2008 (Li et al., 2008a), Staiger, MP, 2006 (Staiger et al., 2006), Witte F, 2008 (Witte et al., 2008), Zhang SX, 2010 (Zhang et al., 2010), Zheng YF, 2014 (Zheng et al., 2014). Among the references with citation burst lasted until 2021, the publication with highest strength was Zhao DW, 2017 (Zhao et al., 2017a), Agarwal S, 2016 (Agarwal et al., 2016), Radha R, 2017 (Radha and Sreekanth, 2017) and Li LY, 2018 (Li et al., 2018).

In Figure 7B, the 10 papers with the strongest citation bursts are five reviews and five original researches. Staiger, MP, 2006 (Staiger et al., 2006), Witte Frank, 2008 (Witte et al., 2008), Witte Frank, 2010 (Witte, 2010) summarize and prospect this industry in the initial stage of magnesium alloy research from the perspective of the properties, biological performance, challenges and future directions of Mg-based biomaterials, which plays a fundamental role in this field. In addition, Zheng YF, 2014 (Zheng et al., 2014) summarized the achievements and problems at the end of the first decade of rapid development of magnesium alloys.

In addition, the corrosion resistance and biocompatibility of Mg-based alloys have always been a problem that researchers are committed to solving. In the initial stage of this field, the mainstream solution to this problem is to test binary alloys *in vitro* and *in vivo*, which is the focus of the five original articles of the 10 references with the strongest citation burst (Witte et al., 2005; Gu et al., 2009a; Zhang et al., 2010).

The most cited articles are often the profound reviews or breakthrough experimental results published by famous scholars in the early stage of the development of the industry, which have received a large number of citations and laid the foundation for the subsequent vigorous development of this field.

Tracing the main research trajectories in a small research domain may be an easy task as scholars do not have to devote great efforts to review a large amount of literature. However, when the research domain grows even larger, the difficulty of tracing the main research trajectories increases significantly. Professor Liu (Liu and Lu, 2012) introduced a quantitative approach, namely, main path analysis, to simplify a large and complicated research domain to one or several main trajectories (paths) consisting of several key nodes and the links, as shown in Figure 7C. These figures were generated by using Pajek software (Mrvar and Batagelj, 2016).

2) A co-cited documents-based clustering analysis.

A co-cited documents-based clustering analysis may present subfields which represent the main research hotspots in this field. Figure 7D presents the clusters of the co-citation network of references**:** “osteogenic differentiation (cluster #0),” “cytotoxicity (cluster #1),” “zinc (cluster #2),” “corrosion resistance (cluster #3),” “mμCT (cluster #4),” “mg-based alloys (cluster #5),” “additive manufacturing (cluster #6),” “microstructure (cluster #7),”“coating (cluster #8),”“nanocomposites (cluster #9),” “corrosion behavior (cluster #10),” “corrosion fatigue (cluster #11),” “calcium phosphate (cluster #12),” “mechanical properties (cluster #13),” “atomic layer deposition (cluster #14),” “mineralized collagen (cluster #15).” The Modularity Q score was 0.6967, >0.5, indicating the network was reasonably divided into loosely coupled clusters. The Weighted Mean silhouette score was 0.8724, more than 0.5, meaning that the homogeneity was acceptable. Index items extracted from articles were used as cluster markers. Apparently, the corrosion of biodegradable Mg-based alloys in human body, containing three dimensions——corrosion resistance, corrosion behavior and corrosion fatigue, is an extremely significant research hotspot. We speculate that many academic achievements will continue to emerge in these subfields for a long time in the future, so as to better improve the biological properties of Mg-based alloy for better clinical application.

### 4.5 Keywords

Keywords in the retrieved publications were extracted and analyzed. The top keywords with the strongest citation bursts were listed in Table 5. We conducted keyword burst analysis to show the keywords whose occurrence frequency increased significantly in a short time (Zou et al., 2018). These keywords can reflect sudden changes and emergent trends in the progress of scientific literature, which can be attributed to new scientific discoveries and technological breakthroughs in this field.

**TABLE 5:** The top keywords with the strongest citation bursts.

Top keywords with the strongest citation bursts					
Keywords	Year	Strength	Begin	End	2005–2021
Stage Ⅰ (2005–2010)					
cartilage/cartilage repair	2005	2.63	**2006**	2014	▂▂▃▃▃▃▃▃▃▃▂▂▂▂▂▂▂
electrochemical behavior	2005	1.14	**2006**	2008	▂▃▃▃▂▂▂▂▂▂▂▂▂▂▂▂▂
calcium phosphate coating/ca-p coating	2005	7.84	**2007**	2018	▂▂▃▃▃▃▃▃▃▃▃▃▃▃▂▂▂
apatite	2005	5.14	**2007**	2012	▂▂▃▃▃▃▃▃▂▂▂▂▂▂▂▂▂
metal matrix composite	2005	4.11	**2007**	2013	▂▂▃▃▃▃▃▃▂▂▂▂▂▂▂▂▂
rat	2005	2.41	**2007**	2011	▂▂▃▃▃▃▃▂▂▂▂▂▂▂▂▂▂
tissue	2005	1.37	**2007**	2015	▂▂▃▃▃▃▃▃▃▃▃▂▂▂▂▂▂
*in vivo* corrosion	2005	25.12	**2008**	2015	▂▂▂▃▃▃▃▃▃▃▃▂▂▂▂▂▂
simulated body fluid	2005	6.39	**2008**	2017	▂▂▂▃▃▃▃▃▃▃▃▃▃▂▂▂▂
ion implantation	2005	6.29	**2008**	2014	▂▂▂▃▃▃▃▃▃▃▂▂▂▂▂▂▂
bone/bone implant application	2005	4.49	**2008**	2010	▂▂▂▃▃▃▂▂▂▂▂▂▂▂▂▂▂
fatigue	2005	2.42	**2008**	2011	▂▂▂▃▃▃▃▂▂▂▂▂▂▂▂▂▂
tensile property	2005	1.68	**2008**	2012	▂▂▂▃▃▃▃▃▂▂▂▂▂▂▂▂▂
stress corrosion cracking	2005	1.29	**2008**	2011	▂▂▂▃▃▃▃▂▂▂▂▂▂▂▂▂▂
az91d	2005	0.76	**2008**	2011	▂▂▂▃▃▃▃▂▂▂▂▂▂▂▂▂▂
pure magnesium	2005	2.8	**2009**	2010	▂▂▂▂▃▃▂▂▂▂▂▂▂▂▂▂▂
surface machining treatment	2005	3.75	**2010**	2014	▂▂▂▂▂▃▃▃▃▃▃▂▂▂▂▂▂
Stage Ⅱ (2011–2015)					
silicate	2005	3.22	**2011**	2014	▂▂▂▂▂▂▃▃▃▃▂▂▂▂▂▂▂
ca/mgca0.8	2005	3.18	**2011**	2013	▂▂▂▂▂▂▃▃▃▂▂▂▂▂▂▂▂
bone resorption	2005	2.7	**2011**	2015	▂▂▂▂▂▂▃▃▃▃▃▂▂▂▂▂▂
animal model	2005	2.16	**2011**	2015	▂▂▂▂▂▂▃▃▃▃▃▂▂▂▂▂▂
amorphous alloy	2005	1.84	**2011**	2014	▂▂▂▂▂▂▃▃▃▃▂▂▂▂▂▂▂
rare earth alloy	2005	1.59	**2011**	2013	▂▂▂▂▂▂▃▃▃▂▂▂▂▂▂▂▂
beta-TCP	2005	1.19	**2011**	2012	▂▂▂▂▂▂▃▃▂▂▂▂▂▂▂▂▂
osseointegration	2005	0.81	**2011**	2012	▂▂▂▂▂▂▃▃▂▂▂▂▂▂▂▂▂
rabbit model	2005	0.81	**2011**	2012	▂▂▂▂▂▂▃▃▂▂▂▂▂▂▂▂▂
vitro/vitro corrosion/vitro degradation	2005	6.4	**2012**	2016	▂▂▂▂▂▂▂▂▃▃▃▃▂▂▂▂▂
zr	2005	6.17	**2012**	2014	▂▂▂▂▂▂▂▃▃▃▂▂▂▂▂▂▂
sol gel coating	2005	0.98	**2012**	2014	▂▂▂▂▂▂▂▃▃▃▂▂▂▂▂▂▂
sr/sr alloy	2005	10.71	**2013**	2019	▂▂▂▂▂▂▂▂▃▃▃▃▃▃▃▂▂
microarc oxidation	2005	6.14	**2013**	2017	▂▂▂▂▂▂▂▂▂▃▃▃▃▂▂▂▂
cell adhesion	2005	4.89	**2013**	2017	▂▂▂▂▂▂▂▂▃▃▃▃▃▂▂▂▂
zn alloy	2005	2.21	**2013**	2018	▂▂▂▂▂▂▂▂▃▃▃▃▃▃▂▂▂
interference screw	2005	1.4	**2013**	2014	▂▂▂▂▂▂▂▂▃▃▂▂▂▂▂▂▂
biomedical application	2005	7.12	**2014**	2018	▂▂▂▂▂▂▂▂▂▃▃▃▃▃▂▂▂
internal fixation	2005	3.12	**2014**	2017	▂▂▂▂▂▂▂▂▂▂▃▃▃▂▂▂▂
aluminum	2005	2.1	**2014**	2015	▂▂▂▂▂▂▂▂▂▃▃▂▂▂▂▂▂
proliferation	2005	1.2	**2014**	2017	▂▂▂▂▂▂▂▂▂▃▃▃▃▂▂▂▂
Stage Ⅲ(2016–2021)					
screw	2005	3.56	**2016**	2021	▂▂▂▂▂▂▂▂▂▂▂▃▃▃▃▃▃
powder metallurgy	2005	1.54	**2016**	2019	▂▂▂▂▂▂▂▂▂▂▂▃▃▃▃▂▂
mesenchymal stem cell	2005	3.3	**2017**	2018	▂▂▂▂▂▂▂▂▂▂▂▂▃▃▂▂▂
high strength	2005	2.27	**2017**	2018	▂▂▂▂▂▂▂▂▂▂▂▂▃▃▂▂▂
osteogenic differentiation	2005	2.02	**2017**	2021	▂▂▂▂▂▂▂▂▂▂▂▂▃▃▃▃▃
heat treatment	2005	1.25	**2017**	2018	▂▂▂▂▂▂▂▂▂▂▂▂▃▃▂▂▂
extrusion	2005	2.34	**2018**	2019	▂▂▂▂▂▂▂▂▂▂▂▂▂▃▃▂▂
fabrication	2005	8.01	**2019**	2021	▂▂▂▂▂▂▂▂▂▂▂▂▂▂▃▃▃
design	2005	5.02	**2019**	2021	▂▂▂▂▂▂▂▂▂▂▂▂▂▂▃▃▃
graphene oxide	2005	4.86	**2019**	2021	▂▂▂▂▂▂▂▂▂▂▂▂▂▂▃▃▃
antibacterial property	2005	4.17	**2019**	2021	▂▂▂▂▂▂▂▂▂▂▂▂▂▂▃▃▃
bioactive glass	2005	4.1	**2019**	2021	▂▂▂▂▂▂▂▂▂▂▂▂▂▂▃▃▃
cytocompatibility	2005	3.83	**2019**	2021	▂▂▂▂▂▂▂▂▂▂▂▂▂▂▃▃▃
nanocomposite	2005	1.05	**2019**	2021	▂▂▂▂▂▂▂▂▂▂▂▂▂▂▃▃▃

The bold values represent the starting time of keywords citation burst.

The keywords with strongest citation burst are divided into three stages according to the starting time, namely stage 1, stage 2 and stage 3. The keywords with strongest burst in stage 1 are “*in vivo* corrosion” (2008–2015), “Ca-P coating” (2007–2013), “simulated body fluid” (2008–2017) and “ion implantation” (2008–2014). The key words that broke out in phase 2 were “Sr/Sr alloy” (2013–2019), “biomedical application” (2014–2018), “Zr” (2012–2014) and “microarc oxidation” (2013–2017). The key words of phase three outbreak are “screw”, “osteogenetic differentiation”, “fabric”, “design”, “graphene oxide”, “antimicrobial property”, “bioactive glass”, “cytocompatibility” and “nanocomposite”. The citation bursts of these keywords continue are still ongoing, indicating that they have received great attention in recent years and may become the research frontiers in the next few years.

## 5 Discussion

Compared with traditional alloys, degradable Mg-based alloys solve the problems of stress shielding and secondary surgery. (Yang et al., 2020).And compared to degradable polymer products such as poly (l-lactic acid) (PLLA), magnesium alloy has a more bone-like mechanical strength and brittleness, which allows it to avoid the risk of re-fracture to a certain extent. (Zhao et al., 2017b).

Systematic bibliometrics analysis including main path analysis, co-cited documents-based clustering analysis, research Frontier analysis can help us review a large number of literatures emerging in this domain since the first article appeared, so as to better understand the development process, hot spots and future trend of the field.

According to the main path analysis and the co-cited documents-based clustering analysis, improving corrosion resistance of Mg-based alloys and revealing the mechanisms underlying the osseointegration and osteoconduction have long been the research hotspot. There are two ways to improve the corrosion resistance of Mg-based alloys: microstructure modification and surface modification.

1) As is shown in Figure 7D, cluster #7 is Microstructure: Microstructure modification includes adjustment of alloy composition, amorphization, grain refinement, heat treatment and improvement of alloy purity. Newly developed microstructures include porous structure, magnesium metal matrix composites (MMCs) and Mg-based bulk metallic glasses (BMGs) (Gu et al., 2010). From Li ZJ, 2008, Gu XN, 2009, Hort N, 2010 to Gu XN, 2012, researchers studied the mechanical properties, corrosion resistance and biocompatibility (cytotoxicity and hemocompatibility) of a variety of different binary Mg- based alloys including Mg-RE alloys (Li et al., 2008b; Gu et al., 2009b; Hort et al., 2010; Gu et al., 2012). They found it was difficult to cast binary Mg-based alloys that could fully meet the requirements. Witte Frank firstly confirmed that the biodegradable magnesium-hydroxyapatite metal matrix composites (MMC-HA) is a cytocompatible biomaterial with adjustable mechanical and corrosive properties. (Witte et al., 2007a).
2) As is shown in Figure 7D, cluster #8 is coating: surface modification includes chemical conversion coatings (Li et al., 2013), micro-arc oxidation coatings, calcium-phosphorus coatings (cluster #12) and biodegradable polymer coatings. Ca-P compound is similar to the inorganic composition of human hard tissue and has excellent biocompatibility. It is one of the most promising biological modified coating materials (Staiger et al., 2006). Though many coatings have been proved to have good biocompatibility, such as the microarc oxidation coating in the core study (Gu et al., 2011), there is still no coating that have been put into clinical application.

The largest cluster in the co-cited documents-based clustering analysis is osteogenic differentiation (cluster #0). From Witte F 2007 to Kraus T, 2012, Chaya A 2015 and Zhang YF 2016, the mechanism in osteoconduction and osseointegration has been a long-standing research hotspot, and in recent years with the application of microfocus CT (mμCT) (mμCT (cluster #4), the research on osseointegration mechanisms has gradually progressed in depth. (Witte et al., 2007b; Witte et al., 2007c; Kraus et al., 2012; Chaya et al., 2015; Zhang et al., 2016).

In addition, analytical methods used in studying the corrosion rate of magnesium alloys *in vivo* and *in vitro* have been stressed by researchers since the early stage. Witte Frank pointed out that the results obtained by adopting *in vitro* measurement could not be directly used to predict *in vivo* corrosion rates (Witte et al., 2006). And in 2008, Witte Frank summarized various analytical methods both *in vitro* and *in vivo*, and prospectively proposed the necessity of establishing effective *in vitro* models that can simulate *in vivo* corrosion, which was deepened by Professor Zhao in 2017 (Witte et al., 2008). Zhao proposed that it was essential to establish a sufficiently complicated animal model that can mimic clinical indications (Zhao et al., 2017b). Sezer summarized the application of computer science in Mg-based alloy research in 2018, including Finite element models (FEM), boundary element method (BEM), CFD model, etc (Sezer et al., 2018). This exhibits a process that is deepening continuously from *in vitro* to *in vivo* and in silico, from animal models to clinical trial.

According to the keyword burst analysis, the research of magnesium alloy is divided into three stages:

1) In the first stage, the research mainly focused on the corrosion and electrochemical mechanism of magnesium alloy *in vivo* (Witte et al., 2005) and *in vitro*, that is, simulated body fluid (Li et al., 2008a), as well as the test of its mechanical properties, including tensile property and stress corrosion cracking (Zhang et al., 2009a). In addition, the research on the optimization of magnesium alloy properties, especially surface machining treatment (Uddin et al., 2015), has also made preliminary progress, including the application of ion implantation (Wan et al., 2008) for surface modification, the application of calcium phosphate coating (Yang et al., 2008) and metal matrix composite (Witte et al., 2007d).
2) In the second stage, the research on alloy composition has become a hot spot, including the addition of various elements: Ca (2011–2013), Zr (2012–2014) (Li et al., 2012), Sr (2013–2019) (Guan et al., 2013), Zn (2013–2018) (Guan et al., 2012), Al (2014–2015) and rare earth elements (2011–2013) (Remennik et al., 2011). In addition, the research on Beta-TCP (Khanra et al., 2010) and the application of sol gel coating (Tang et al., 2013) and micro-arc oxidation layer (Gao et al., 2011) also further improves the properties of magnesium alloy. The research on the cytocompatibility of the magnesium alloy has also been further deepened, including its role in bone resorption, osseointegration (Castellani et al., 2011) and cell adhesion (Zhang et al., 2009b). At this stage, the clinical application of magnesium alloy has been gradually emphasized by scholars, including its application in orthopedic surgery as an interference screw (Ezechieli et al., 2016) in internal fixation.
3) The keywords emerging in the third stage represent advanced research directions and hotspots, including the application of mesenchymal stem cells combined with magnesium alloy in orthopedic therapy and the application of graphene oxide (Zhao et al., 2018), bioactive glass (Yin et al., 2019) and nanocomposite (Mousa et al., 2018) in magnesium alloy manufacturing. In addition, the fabrication and design strategies of magnesium alloy production and manufacturing have also been emphasized by researchers, including the application of additive manufacturing (Wang et al., 2016) to produce biocompatible metals to provide patient specific, site specific, morphology specific and function specific implants.

Methods to improve the performance of magnesium based orthopedic implants in three stages are summarized as follows, which is also the main means to solve the inherent problems of magnesium alloys at present**:**

1 Alloying

1) Binary Mg-based alloys: Mg-Al, Mg-Mn, Mg-Zn, Mg-Ca, Mg-Sr alloys, etc.
2) Ternary Mg-based alloys: Mg-Zn-Ca, Mg-Zn-Sr alloys, etc.
3) Quaternary magnesium alloy: Mg-Zn-Zr-Y, Mg--Zn--Sn--Sr alloys, etc.

2 Mg-based composites: Mg-Hydroxyapatite composites, Mg-Bioactive glass composites, etc.

3 Coatings: HA coatings, Micro-arc oxidation (MAO) coating; Chemical conversion coating; Polymeric coatings; fluoride coatings; Bone morphogenetic proteins (BMP) coatings; Self-assembly Monolayer (SAM) coatings, etc.

4 Methods to fabricate porous implants

1) Powder metallurgy (PM), Titanium wire space holder (TWSH) technique.
2) Additive manufacturing (AM) techniques**:** Powder bed fused (PBF) technique, Wire arc additive manufacturing (WAAM), Three-Dimensional (3D) jet printing, Paste extrusion deposition (PED).
3) Electric discharge drilling (EDD).
4) Melting techniques.

According to our keyword analysis, screws have a stronger citation burst in recent years, which is inseparable from the increase in clinical trials and *in vivo* testing of magnesium alloys in recent years.

In 2015, Ajou University Hospital (Suwon, Korea) conducted a 1-year postoperative follow-up of magnesium alloy screws in surgical fixation of distal radius. It was found that magnesium alloy screws have excellent biocompatibility and bone integrity (Lee et al., 2016). At Hannover Medical University, a clinical study on the application of Mg-Y-RE-Zr screws produced by Syntellix company in Germany in 2010 confirmed that it had a good effect in hallux valgus surgery (Windhagen et al., 2013). In addition, the Medical College of Dalian University from China applied pure magnesium screws to free iliac bone transplantation for femoral head necrosis in a randomized controlled trial (Zhao et al., 2016), and achieved significantly better osteogenic performance than the control group. These findings preliminarily confirmed the osteogenesis of magnesium alloy screws in the non-weight-bearing area. The performance of magnesium alloy implants in the load-bearing area and high torsion area needs more tests to confirm. With more clinical trials, the research of biodegradable magnesium alloy implants will be further promoted.

According to the above discussion based on reference and keyword analysis, we clearly show the global research trends and progress in this field. Researchers will be committed to optimizing the microstructure by improving manufacturing methods and process parameters in the future. In addition, the research on alloying, coating and Mg-bioglass composites will still be a hot spot for a long time. If researchers are committed to the research to improve the performance of implants and the research on clinical translational applications, they may achieve greater breakthroughs.

### 5.1 Managerial implications of the study

This study shows that the research on biodegradable magnesium alloys is still in full swing, and the citation and keyword bursts are used to speculate on the future hot spots and research trends. This study provides managerial implication for academia, industry, scholars and medical researchers. Researchers and relevant institutions can utilize our conclusions and the massive information contained in tables and pictures according to their own requirements. This study helps researchers in industry and medicine clarify their own research direction faster, choose scholars and institutions that can cooperate, so as to accelerate the research progress of medical magnesium alloys and obtain stable clinically available medical magnesium alloy products as soon as possible.

### 5.2 Limitations

There are certain limitations in our study. Firstly, in our study, we only searched the Web of Science Core Collection (WoSCC) and did not incorporate other databases, such as PubMed, Scopus or Embase. However, it may be unscientific to merge and analyze the data from multiple databases, because different databases have different measurement of citation frequency counting and classification of publications (Wu et al., 2021/10; Wu et al., 2021).

Besides, there may exist differences between the real world and the present results. For example, the latest publications may not be emphasized enough because of lower citation frequency due to the lack of temporal accumulation (Sangwal, 2014).

Therefore, we need to pay continued attention to non-English studies as well as the latest high-quality studies in our daily scientific research work.

## 6 Conclusion

This is the first bibliometric analysis to comprehensively evaluate the general aspects and future trends in the field of the biodegradable Mg-based alloy for orthopedic applications.

We collected 1921 articles from 2005 to 2021 from Web of Science database to carry out this research work. The paper “The development of binary Mg-Ca alloys for use as biodegradable materials within bone” by Li et al. (2008) was the article with the strongest citation burst. The paper “Magnesium and its alloys as orthopedic biomaterials: a review” is the most cited article. The most influential journals in this field are Acta Biomaterialia and Biomaterials, with 16,511 and 12,314 total citations, respectively. Zheng Yufeng ranked first with 85 articles and 7,421 citations, followed by Witte Frank and Yang Ke. The corresponding institutions with the highest production are Chinese Academy of Sciences, Peking University and Shanghai Jiao Tong University.

According to the keyword burst analysis, we divide the development and application of biodegradable magnesium alloys into three stages: the first is the research of mechanical properties and degradation characteristics of magnesium alloys, the second is the research on the composition and coating of magnesium alloys, and this research hotspot has continued to the present, and then enter the “pre-clinical application stage”, focusing more on clinical use, including the application of nano materials and mesenchymal stem cells, exploration of angiogenesis promotion and antibacterial ability, etc.

It is observed that the research on biodegradable magnesium alloy has been gradually deepened, and scientists and clinical researchers have tried to use it for clinical use, but there is still a gap between its basic research and development and clinical practical application. So far, there is still no biodegradable magnesium alloy product that can be stably used in clinic. This bibliometric research shows a broad field for future young researchers, which helps them shorten the threshold of entering this field and quickly comprehend the development status and pattern.
